# Risk Factors and Mental Health Status in Patients With Non-Tuberculous Mycobacterial Lung Disease: A Single Center Retrospective Study

**DOI:** 10.3389/fpubh.2022.912651

**Published:** 2022-08-01

**Authors:** Zhangyan Zhao, Huiliang Hu, Mei Wang, Feng Li, Haicheng Tang

**Affiliations:** Department of Respiratory and Critical Care Medicine, Shanghai Public Health Clinical Center, Fudan University, Shanghai, China

**Keywords:** NTM, NTM lung disease, mental health, influencing factors, anxiety

## Abstract

According to the existing data, the incidence and prevalence of non-tuberculous mycobacteria (NTM) are increasing worldwide. The risk factors and mental health status of patients with NTM lung disease are important and deserve our attention. A total of 180 patients with NTM lung disease hospitalized from January 2018 to December 2021 were selected as the NTM group, and 90 patients with non-severe community-acquired pneumonia (CAP) who were hospitalized during the same period were selected as the control group. The Symptom Checklist-90 (SCL-90) was used to assess the mental health status of the patients. The data were analyzed using descriptive statistics, logistic regression, and receiver operating characteristic (ROC) curves. There were no significant differences in age, sex, marital status, or smoking history between the two groups (*p* > 0.05), but there were significant differences in educational level, underlying diseases, occupation, living environment, and body mass index (BMI) (*p* < 0.01). According to the classification of basic diseases, bronchiectasis was found in 82 (45.6%) patients, followed by hypertension in 66 (36.7%) patients, and chronic obstructive pulmonary disease (COPD) in 39 (21.7%) patients. The NTM strains were identified *M. intercelleulare* caused 41 cases (22.8%), followed by *Mycobacterium avium* and *Mycobacterium gordonae*, each with 35 cases (19.4%), and *Mycobacterium abscessus* with 32 cases (17.8%). The SCL-90 found that 160 (88.9%) of 180 patients with NTM lung disease had developed mental health problems, among which the four highest-scoring factors were anxiety (ANX: 29.4%), depression (DEPR: 18.8%), sleep and diet (SD: 16.9%), and somatization (SOM: 11.3%). Through multivariate logistic regression analysis, it was found that educational level, underlying diseases, living environment, and BMI were independent risk factors for the occurrence of NTM lung disease (*p* < 0.01). The Hosmer–Lemeshow test was used to check the model's fitness. The ROC curve showed that the area under the curve (AUC) was 0.896, the sensitivity was 83.3%, and the specificity was 85.6%. Patients with NTM lung disease have many risk factors and prominent mental health problems that may require interventions during the process of clinical diagnosis and treatment.

## Introduction

Non-tuberculous mycobacteria (NTM) is a general term for a large group of mycobacteria that does not include the *Mycobacterium tuberculosis* complex and *Mycobacterium leprae*. NTM exists widely in natural environments, such as water, soil, and dust, and can infect humans and some animals. To date, more than 190 species of NTM have been discovered. Most of them are parasitic bacteria, and only a few are pathogenic to the human body, which are opportunistic pathogens (1–4). NTM disease refers to the infection of the human body with NTM, which causes lesions in related tissues and organs. According to the existing data, the incidence and prevalence of NTM are increasing worldwide, and in some countries and regions, they even exceed the incidence and prevalence of tuberculosis (TB) (1, 5). NTM exploits opportunities in the human body. A large number of cases of sexually transmitted NTM infections have been found, and they have become an important group of pathogens that threaten human health and life (6–8). There are no epidemiological survey data specifically on large-scale NTM disease in China, but previous epidemiological survey data on TB in China showed that the isolation rate of NTM increased from 4.3% in 1979 to 11.1% in 2000 and 22.9% in 2010, reflecting a significant upward trend in NTM disease in China (9).

Non-tuberculous mycobacteria lung infection is the most common NTM disease, accounting for ~70–80% of NTM infections. There are no specific data on this aspect in China (8, 10, 11). In recent years, the NTM strains that cause lung lesions have undergone certain changes. The main strains are *Mycobacterium avium complex (MAC), Mycobacterium abscessus (M. abscessus), Mycobacterium kansasii (M. kansasii), Mycobacterium malmo (M. malmo), and Mycobacterium vaccinia (M. vaccinia)*, followed by *Mycobacterium chelae (M. chelae), Mycobacterium fortuitum (M. fortuitum), Mycobacterium haemophilus (M. haemophilus)*, etc., and two or more NTM strains can also infect the same individual at the same time (12, 13).

Non-tuberculous mycobacteria lung disease is a chronic disease that can occur at any age. The prevalence in women is higher than that in men, and most of them are older adults, usually postmenopausal women (14). Most patients have underlying lung diseases, such as chronic obstructive pulmonary disease (COPD), bronchiectasis, cystic pulmonary fibrosis, etc., and some patients have scoliosis, mitral valve prolapse, etc. (15, 16). Some patients can also be infected with NTM lung disease after organ transplantation and mechanical ventilation (17, 18).

Once a patient is infected with NTM lung disease, in addition to the torment of the disease, they also face pain caused by long-term medication needs and adverse drug reactions, an increased economic burden, and an increased risk of mental illness. Our hospital is a designated hospital for infectious diseases in Shanghai. It treats a large number of patients with TB and non-TB infections every year. The routine evaluation of patients' mental health enables the majority of the medical staff to deepen their understanding of NTM lung disease, improving the level of diagnosis and prevention of NTM lung disease and reducing its harm to human life and health.

## Materials and Methods

### Study Design and Sample

**Figure 1:**
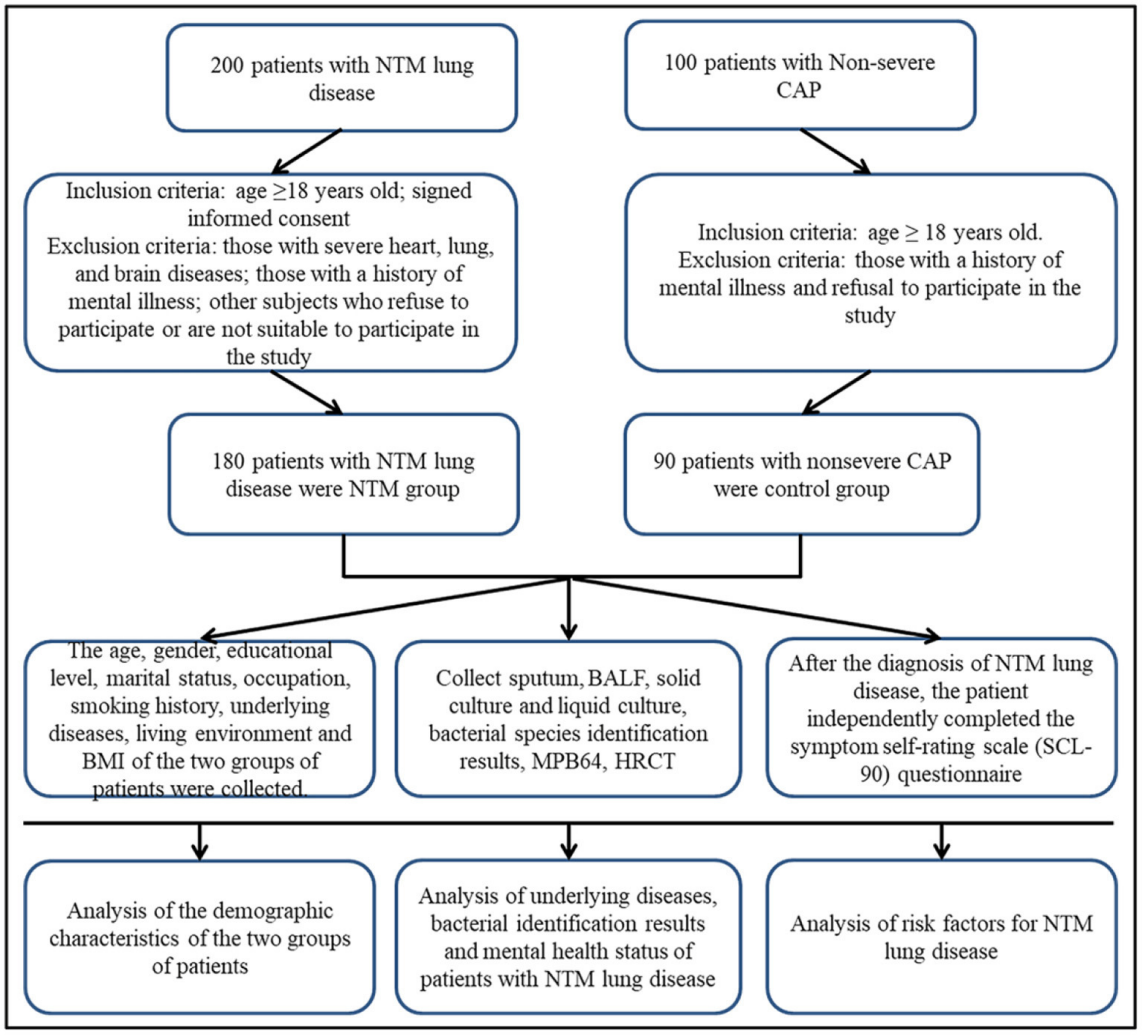
Flow diagram of the study. NTM, non-tuberculous mycobacteria; CAP, community-acquired pneumonia; HRCT, High-resolution CT; BALF, bronchoalveolar lavage fluid.

A total of 200 NTM lung disease patients who were hospitalized at Shanghai Public Health Clinical Center from January 2018 to December 2021 were selected, and 180 patients were finally included in the NTM group. The **inclusion criteria were:** met the diagnostic criteria for NTM lung disease in the “Expert Consensus on the Diagnosis and Treatment of Non-tuberculous Mycobacteriosis” (1); age ≥ 18 years old; and signed informed consent. The **exclusion criteria were:** those with severe heart, lung, and brain diseases; those with a history of mental illness; refusal to participate; subjects who were not fit enough to participate in research. A total of 100 patients with non-severe community-acquired pneumonia (CAP) who were hospitalized during the same period were selected, and 90 patients were included in the control group. The inclusion criteria were: age ≥ 18 years old; the diagnosis of non-severe CAP complied with the “Guidelines for the Diagnosis and Treatment of Community-Acquired Pneumonia in Chinese Adults (2016 Edition)” (19). The exclusion criteria were: those with a history of mental illness and refusal to participate in the study (Figure 1).

### Data Collection

The demographic characteristics of the enrolled patients were collected, such as age, sex, education level, marital status, occupation, smoking history, underlying diseases, living environment, and body mass index (BMI), and the results of the NTM strain identification and the SCL-90 questionnaire were collected. This research was conducted in accordance with the Declaration of Helsinki. The data were accurate and reliable and approved by the ethics review committee of the Shanghai Public Health Clinical Center. All patients provided informed consent, and the questionnaires were anonymous and returned after completion.

#### Sputum Specimen Collection

Specimens were collected before the administration of antibacterial drugs by collecting morning phlegm after fasting for at least 1–2 h. The patient was asked to gargle three times with 0.1% spirulina before expelling loose sputum, and then they gargled once with water before coughing up the deep sputum, which was placed in a sterile container and sent for inspection. For patients with little or no sputum, 3% saline was used to induce sputum by atomization, and the method of collecting sputum after atomization was as described previously. After collection and labeling, the specimens were placed at room temperature and sent to the microbiology laboratory within 2 h. Sputum was collected at 24-h intervals for three consecutive days.

#### Collection of Bronchoalveolar Lavage Fluid

Before bronchoalveolar lavage (BAL), the patient's clinical status was evaluated and the risk of bleeding and contraindications to bronchoscopy were taken into account. The patient's vital signs were monitored, the airway was fully anesthetized, and BAL was performed before biopsy and brushing. The lesion site for BAL was selected, and normal saline was injected in divided doses, 20–50 ml each time. Immediately after injecting normal saline, appropriate negative pressure suction was used to obtain the BALF, and the total recovery rate was ≥30%. Specimens were collected in sterile containers. The first tube of the recovered solution was discarded and the second tube was sent for routine etiological examination. The sample was 10–20 ml, labeled and placed at room temperature, and then sent to the microbiology laboratory within 2 h.

#### Bacterial Identification

The bacterial species were identified by the PCR-reverse dot hybridization method. The detection method was as follows: DNA extraction from sputum samples-PCR amplification-hybridization-washing membrane-color development-observation results. A Mycobacterium species identification gene detection kit (25 copies/box), production batch number: 21M11001 M, company: Yaneng BIOscience (Shenzhen) Co., Ltd. Gene amplification instrument and molecular hybridization box: FinePCR YN-H16 were used following the manufacturer's instructions.

#### Symptom Self-Rating Scale

The SCL-90 has proven to be a useful tool for assessing a wide range of psychological problems and psychopathological symptoms. The scale consists of 90 items in 9 dimensions. Each item is scored on a scale of 1–5, representing asymptomatic to severe symptoms. The main symptom dimensions assessed were as follows: obsessive-compulsive symptoms (OCS), interpersonal sensitivity (INTS), somatization (SOM), depression (DEPR), hostility (HOS), anxiety (ANX), paranoid ideation (PARI), phobias anxiety (PHOA), sleep and diet (SD), and psychosis (PSY). Ten factors were used to reflect the psychological symptoms of the 10 aspects. The scale of each factor is as follows: 1–1.99 points indicate no mental health problems; 2–2.99 points indicate mild mental health problems; 3–3.99 points indicate moderate mental health problems; 4–4.99 points indicate relatively serious mental health problems; and 5 points indicate serious mental health problems. According to the national norm, if the total score is more than 160 points, the number of positive items is more than 43 points, or if the score of any factor is more than 2 points, it is regarded as positive, and further examination is required (20).

#### Quality Control

All patients underwent sputum examination after admission, and some patients without sputum underwent BALF examination. The patients were subjected to a standardized collection of sputum specimens, bronchoscopy to collect bronchoalveolar lavage fluid (BALF), bacterial identification, routine sputum acid-fast bacilli smear examination, liquid culture combined with solid culture, negative MPB64 antigen test to rule out TB, high-resolution CT (HRCT) scan of the chest, and clinical specimens were tested within 2 h of collection. Two sputum cultures or one BALF culture of the same species can confirm the diagnosis of NTM. The self-rating symptom scale (SCL-90) questionnaire was anonymous. The patient completed the questionnaire after NTM lung disease was diagnosed. Some patients completed the questionnaire during the outpatient follow-up under the supervision and guidance of a doctor.

### Statistical Analysis

We used SPSS 21.0 software to analyze the data. The measurement data are expressed as (x ± s) and comparisons between groups were made by *t*-tests or the chi-square test; the count data are expressed as *n* (%). Multivariate logistic regression was used to identify the NTM lung disease risk factors. Receiver operating characteristic (ROC) curves were drawn. The value of *p* < 0.05 indicates that the difference is statistically significant.

## Results

### Sample Description

The demographic characteristics of the two groups were analyzed. The NTM group had 68 men and 112 women, aged 34–78 years, with an average age of 60.15 ± 3.28 years; the control group had 42 men and 48 women, aged 30–73 years, with an average age of 58.32 ± 6.42 years. There were no significant differences in age, sex, marital status, or smoking history between the two groups (*p* > 0.05, Table 1).

**Table 1:** Comparison of the sociodemographic characteristics of the two groups of participants.

**Characteristics**	***n* = 270**	**NTM group** **(*n* = 180)**	**Control group** **(*n* = 90)**	***χ^2^***	***p*-Value**
Age				0.030	0.863
≤ 50 yrs	128	86	42		
>50 yr	142	94	48		
Gender				1.964	0.161
Male	110	68	42		
Female	160	112	48		
Education level				12.157	**0.002**
Junior high school	80	65	15		
High school	113	72	41		
The University	77	43	34		
Marital status				1.795	0.408
Unmarried	16	10	6		
Married	232	158	74		
Divorced/Widowed	22	12	10		
Underlying disease				89.896	**0.000**
Yes	186	158	28		
No	84	22	62		
Occupation				9.645	**0.008**
Farmer	77	62	15		
Freelance	95	60	35		
Permanent job	98	58	40		
Living environment				20.972	**0.000**
Rural	100	82	18		
Suburban	88	57	31		
Urban	82	41	41		
Smoking history				0.030	0.862
Yes	118	78	40		
No	152	102	50		
BMI				39.725	**0.000**
Low	125	105	20		
Normal	83	51	32		
High	62	24	38		

*The bold values means that the p value is statistically significant (p <0.05)*.

### Influencing Factors and Common Strains of NTM Lung Disease

There were significant differences in education level, underlying diseases, occupation, living environment, and BMI between the two groups (*p* < 0.01; Table 1). According to the classification of basic diseases, bronchiectasis occurred in 82 (45.6%) patients, followed by hypertension in 66 (36.7%) patients, and COPD in 39 (21.7%) patients (Table 2). The NTM strain identification showed that *M. intercelleulare* had 41 cases (22.8%), followed by *M. avium* and *Mycobacterium gordonae*, each with 35 cases (19.4%), and *M. abscessus* with 32 cases (17.8%) (Table 3).

**Table 2:** Classification of the underlying disease in patients with non-tuberculous mycobacteria (NTM) lung disease.

**Classification**	***n***	**%**
Bronchiectasis	82	45.6
Tuberculosis	20	11.1
Tumor	12	6.7
Pneumoconiosis	15	8.3
Hypertension	66	36.7
Connective tissue disease	12	6.7
Diabetes	28	15.6
COPD	39	21.7
HIV	19	10.6
Others	22	12.2

**Table 3:** Bacterial identification in patients with NTM lung disease.

**Bacterial identification**	***n* = 180**	**%**
*M. avium*	35	19.4
*M. kansasii*	10	5.6
*M. malmoense*	3	1.7
*M. haemophilum*	4	2.2
*M. abscessus*	32	17.8
*M. smegmatis*	3	1.7
*M. colombiense*	3	1.7
*M. maeseillense*	5	2.8
*M. chelonae*	2	1.1
*M. intercelleulare*	41	22.8
*M. vaccae*	1	0.6
*M. gordonae*	35	19.4
*M. fortuitum*	2	1.1
Others	4	2.2

### SCL-90 Assessment of Mental Health in Patients With NTM Lung Disease

As shown in Table 4, in this study, the statistical results of the SCL-90 on the mental health of patients in the NTM group found that a total of 33 participants scored higher than 160 points, accounting for 20.6%, and 62 participants had a total of positive items higher than 43 points (38.8%). It was found that forty-five participants had factor scores higher than 2 (28.1%). Among all factors, OCS was 1.54 ± 0.27, SOM was 1.87 ± 0.29, HOS was 1.63 ± 0.41, DEPR was 2.12 ± 0.27, INTS was 1.71 ± 0.33, PHOA was 1.41 ± 0.48, ANX was 2.53 ± 0.36, PARI was 1.50 ± 0.32, sleep and diet were 1.98 ± 0.32, and PSY was 1.37 ± 0.26. We found that the four highest-scoring factors were ANX (29.4%), DEPR (18.8%), sleep and diet (16.9%), and SOM (11.3%).

**Table 4:** The symptom checklist-90 (SCL-90) assessment of the mental health of patients with NTM lung disease.

**Factors**	***n***	**NTM group (*****n*** **=** **160)**					
		**Mild (96)**	**Moderate (53)**	**Severe (9)**	**Serious (2)**	**Average**	**%**
OCS	7	4	3	0	0	1.54 ± 0.27	4.4
SOM	18	11	6	1	0	1.87 ± 0.29	11.3
HOS	8	6	2	0	0	1.63 ± 0.41	5.0
DEPR	30	19	8	2	1	2.12 ± 0.27	18.8
INTS	10	7	2	1	0	1.71 ± 0.33	6.3
PHOA	4	2	2	0	0	1.41 ± 0.48	2.5
ANX	47	25	18	3	1	2.53 ± 0.36	29.4
PARI	6	5	1	0	0	1.50 ± 0.32	3.8
SD	27	15	10	2	0	1.98 ± 0.32	16.9
PSY	3	2	1	0	0	1.37 ± 0.26	1.9

### Independent Risk Factors for NTM Lung Disease

**Figure 2:**
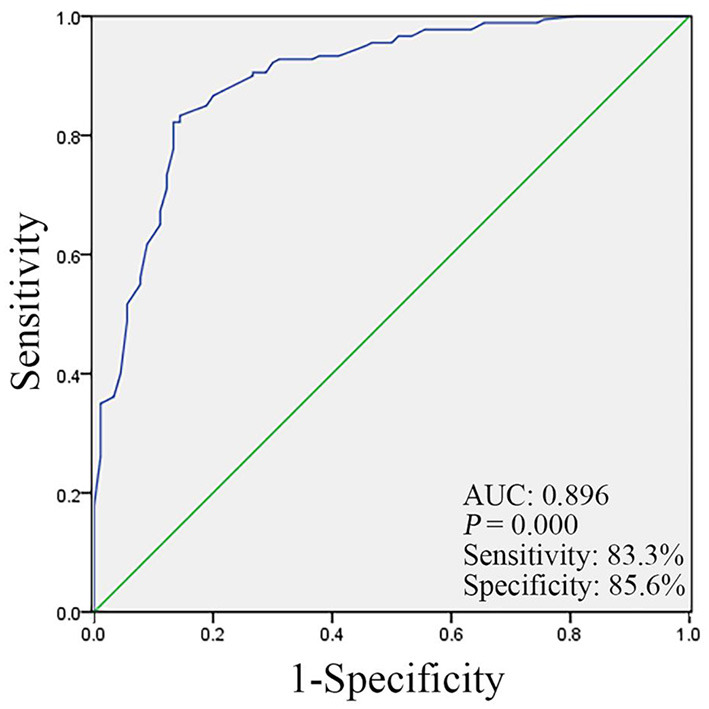
The receiver operating characteristic (ROC) curve analysis for the education level, underlying diseases, living environment, and body mass index (BMI) value in the NTM group and control group.

Multivariate logistic regression analysis of various factors that may affect patients with NTM lung disease found that educational level (*p* = 0.003, 95% CI 0.066–0.581), underlying diseases (*p* = 0.000, 95% CI 9.076–47.419), living environment (*p* = 0.001, 95% CI 0.079–0.530), and BMI (*p* = 0.000, 95% CI 0.037–0.262) were independent risk factors for NTM lung disease (Table 5). The Hosmer–Lemeshow test shows that the model fits the predictors well. We included four important variables (educational level, underlying disease, living environment, and BMI) in logistic regression to calculate probabilities and made ROC curves based on the obtained probabilities. The ROC curve indicated an area under the curve (AUC) of 0.896. A cut-off score of 0.689 yielded the best sensitivity (83.3%) and specificity (85.6%) (Figure 2).

**Table 5:** Logistic regression analysis of high-risk factors in patients with NTM lung disease.

**Characteristics**	**B**	**S.E**,	**Wals**	**Sig**.	**Exp (B)**	**95% CI**
Age	−0.405	0.381	1.131	0.287	0.667	0.316–1.407
Gender	0.283	0.401	0.498	0.480	1.327	0.605–2.910
Education level	1.630	0.554	8.637	**0.003**	5.101	0.066–0.581
Marital status	0.149	0.931	0.026	0.873	1.161	0.139–5.341
Underlying disease	3.032	0.422	51.682	**0.000**	20.745	9.076–47.419
Occupation	0.474	0.498	0.905	0.341	1.606	0.235–1.653
Living Environment	1.589	0.487	10.656	**0.001**	4.900	0.079–0.530
Smoking history	0.288	0.382	0.569	0.451	1.334	0.631–2.820
BMI	2.314	0.498	21.597	**0.000**	10.115	0.037–0.262

*The bold values means that the p value is statistically significant (p <0.05).*

*CI, confidence interval; SE, standard error*.

## Discussion

The individual size of NTM is (0.2–0.6) × (1.0–10) μm, and it is positive for Gram staining. Generally, it lacks spores, flagella, and other structures and does not produce endotoxin or exotoxin. Its pathogenicity and bacterial components are related. Previous researchers believed that NTM were very weak pathogenic bacteria, and the possibility of disease was relatively low (21, 22). With its increasing incidence, in-depth research on the disease has found that NTM is an opportunistic pathogen that can harm the health of humans and animals, and some non-tuberculous mycobacterial infections are quite serious.

The ways in which NTM enter the body include infections of the respiratory tract, digestive tract, and skin contact (23, 24). The lung is the organ most frequently affected by non-tuberculous mycobacterial infection, and it has been reported that the proportion of pulmonary disease can reach 95%. The pathogenesis of NTM and *M. tuberculosis* is very similar (25), and their clinical manifestations and imaging features are also very similar.

The reasons for the increased incidence of NTM disease are unclear but may be related to increased awareness of NTM disease among clinicians, an aging population, an increase in the immunosuppressed population, long-term medication use, environmental exposures, improved laboratory testing methods, and a certain relationship with human-to-human transmission (1, 3, 4, 26–30). The incidence of NTM is closely related to age. With increasing age, the incidence further increases, and there are differences in the distribution of sexes (5, 8, 26, 27). In Europe, the incidence is often high in men and young people, while in North America, women and the older adults have a high incidence, especially postmenopausal women with low BMI and a tall and thin body shape (26, 31).

This study aimed to analyze the data of patients with NTM lung disease admitted to our hospital. The presence of NTM lung disease was related to the patient's education level, underlying diseases, occupation, living environment, and BMI. We believe that education level itself is not a significant risk factor for NTM lung disease, but a lower education level may be correlated with a worse occupational and residential environment, which may be a risk factor for NTM lung disease. Similarly, a low education level may be correlated with a lower income level and poor living conditions of patients, which is also related to a poor nutritional status, resulting in a lower than normal BMI, which is a significant risk factor for NTM lung disease.

Previous studies have found that people with underlying lung diseases, such as bronchiectasis, pneumoconiosis, TB, etc., or HIV infection, tumors, organ transplantation, and other diseases are more likely to become sick (32–34). This study also found that underlying diseases are closely related to the occurrence of NTM lung disease, most commonly bronchiectasis, followed by hypertension and COPD. The Chinese literature reported that there are more men than women with NTM disease, and 40% (52/130) are aged 60 years and over (5), while the ratio of men to women in patients with bronchiectasis combined with NTM disease is 1:1.9, and middle-aged and older adult women are the most common (35).

Non-tuberculous mycobacteria can be divided into fast-growing NTM and slow-growing NTM according to their growth speed. Slow-growing NTM can be divided into photochromogenic NTM, dark-chromogenic NTM, and a chromogenic NTM according to their colony characteristics. To date, more than 190 species of NTM species have been found. In this study, *M. intercelleulare* caused 41 cases (22.8%), followed by *M. avium* and *M. gordonae*, each with 35 cases (19.4%), and *M. abscessus* had 32 cases (17.8%).

There are many reasons for the psychological problems of patients with NTM lung disease. Patients with NTM lung disease have a long course of the disease. During the course of the disease, they will experience repeated acute exacerbations and be repeatedly hospitalized. Long-term medication is needed. There are inferiority complexes and psychological shadows, indifferent interpersonal relationships, and some even lose the ability to work, increasing their economic burden and leading to depression, anxiety, mania, and sleep disorders. The SCL-90 questionnaire can comprehensively evaluate the mental health status of patients and has good validity and reliability. In this study, after the SCL-90 questionnaire was tested in patients with NTM lung disease, it was observed that patients with NTM lung disease did have some mental health problems. The four highest-scoring factors were ANX (29.4%), DEPR (18.8%), sleep and diet (16.9%), and SOM (11.3%). Overall, the psychological problems of patients with NTM lung disease are many and complex and deserve social attention.

In conclusion, education level, underlying diseases, living environment, and BMI are independent risk factors for NTM lung disease. Similar to other chronic diseases, NTM lung disease is associated with physical and mental health problems. It is necessary to target patients at risk during the process of clinical diagnosis and treatment. There is a need to pay attention to mental health problems and make more efforts to prevent the occurrence of NTM lung disease and improve the quality of life of the patients.

## Limitations

Several limitations should be considered in this study, including that it is a sample from a single hospital with a limited number of patients. In future studies, patients should be studied in multiple hospitals to better generalize the findings and compare the differences. Finally, social support should also be provided for patients with NTM lung disease.

## Relevance for Clinical Practice

The results of this study provide information about patients with NTM lung disease who have risk factors and mental health disorders. This information may help doctors and nurses or hospital authorities take necessary measures to provide support and interventions for the mental health of patients with NTM lung disease, who are more likely to develop psychological problems than other types of patients.

## Data Availability Statement

The original contributions presented in the study are included in the article/Supplementary Material, further inquiries can be directed to the corresponding authors.

## Ethics Statement

The studies involving human participants were reviewed and approved by the Ethics Committee of the shanghai public health clinical center. The patients/participants provided their written informed consent to participate in this study. Written informed consent was obtained from the individual(s) for the publication of any potentially identifiable images or data included in this article.

## Author Contributions

ZZ designed the study and drafted the manuscript. MW and HH collected the clinical data and processed statistical data. FL analyzed and interpreted the data. HT designed, supervised the study, and revised the manuscript. All authors read and approved the final version of the manuscript.

## Funding

Project supported by the Shanghai Municipal Health Commission (Grant No. 202040332—HT), the Shanghai Municipal Science and Technology Major Project (Grant No. ZD2021CY001—FL), the Shanghai Science and Technology Commission (Grant Nos. 20Z11901002 and 21Y11901700—FL), and the Shanghai Public Health Clinical Center (Grant No. KY-GW-2021-16—HT).

## Conflict of Interest

The authors declare that the research was conducted in the absence of any commercial or financial relationships that could be construed as a potential conflict of interest.

## Publisher's Note

All claims expressed in this article are solely those of the authors and do not necessarily represent those of their affiliated organizations, or those of the publisher, the editors and the reviewers. Any product that may be evaluated in this article, or claim that may be made by its manufacturer, is not guaranteed or endorsed by the publisher.
