# Supplementary material for: Risk Factors and Mental Health Status in Patients With Non-Tuberculous Mycobacterial Lung Disease: A Single Center Retrospective Study
Source: Front Public Health. 2022 Aug 1;10:912651. doi: 10.3389/fpubh.2022.912651 (PMC9376471; doi:10.3389/fpubh.2022.912651)
Supplement: Supplementary file 1 [file Table_1.docx]

| **= Hosmer and Lemeshow test =** | | | |
| --- | --- | --- | --- |
| step | Chi-square | df | Sig. |
| 1 | 6.666 | 8 | .573 |
